# The Clinical Relevance of Artificial Intelligence in Migraine

**DOI:** 10.3390/brainsci14010085

**Published:** 2024-01-16

**Authors:** Angelo Torrente, Simona Maccora, Francesco Prinzi, Paolo Alonge, Laura Pilati, Antonino Lupica, Vincenzo Di Stefano, Cecilia Camarda, Salvatore Vitabile, Filippo Brighina

**Affiliations:** 1 Department of Biomedicine, Neuroscience and Advanced Diagnostics (Bi.N.D.), University of Palermo, 90127 Palermo, Italy; angelo.torrente@unipa.it (A.T.); simonamaccora1@gmail.com (S.M.); francesco.prinzi@unipa.it (F.P.); paolo.alonge01@unipa.it (P.A.); laura.pilati.91@gmail.com (L.P.); antlupica@gmail.com (A.L.); vincenzo19689@gmail.com (V.D.S.); cecilia.camarda@unipa.it (C.C.); salvatore.vitabile@unipa.it (S.V.); 2 Neurology Unit, ARNAS Civico di Cristina and Benfratelli Hospitals, 90127 Palermo, Italy; 3 Department of Computer Science and Technology, University of Cambridge, Cambridge CB2 1TN, UK; 4 Neurology and Stroke Unit, P.O. “S. Antonio Abate”, 91016 Trapani, Italy

**Keywords:** migraine, migraine diagnosis, migraine management, migraine attack prediction, artificial intelligence, machine learning, personalised medicine

## Abstract

Migraine is a burdensome neurological disorder that still lacks clear and easily accessible diagnostic biomarkers. Furthermore, a straightforward pathway is hard to find for migraineurs’ management, so the search for response predictors has become urgent. Nowadays, artificial intelligence (AI) has pervaded almost every aspect of our lives, and medicine has not been missed. Its applications are nearly limitless, and the ability to use machine learning approaches has given researchers a chance to give huge amounts of data new insights. When it comes to migraine, AI may play a fundamental role, helping clinicians and patients in many ways. For example, AI-based models can increase diagnostic accuracy, especially for non-headache specialists, and may help in correctly classifying the different groups of patients. Moreover, AI models analysing brain imaging studies reveal promising results in identifying disease biomarkers. Regarding migraine management, AI applications showed value in identifying outcome measures, the best treatment choices, and therapy response prediction. In the present review, the authors introduce the various and most recent clinical applications of AI regarding migraine.

## 1. Introduction

Migraine is a primary headache typically presenting with recurring episodes of moderate-to-severe unilateral throbbing pain that may usually last from 4 to 72 h [1]. The episodes in some patients may be preceded or accompanied by transient focal neurological symptoms (i.e., aura), configuring migraine with aura (MwA), a subpopulation quite peculiar compared to migraine without aura (MwoA) patients. Migraine is one of the most frequent neurological disorders, with an estimated prevalence that goes up to 15% of the worldwide population [2]. Due to its disabling nature, it represents in many ways a societal problem since it entails very high direct and indirect costs on a large scale [3]. Based on the presentation of pain episodes, migraine can be classified as chronic (CM—i.e., headache for at least 15 days per month with 8 or more showing migraine features for at least 3 months) or episodic (EM—i.e., if the abovementioned criteria are not met) [1]. Moreover, one of the main issues is that there is still a consistent number of patients who do not receive a diagnosis, even because of the very high prevalence of the disease. In addition, many patients are misdiagnosed due to the difficulty of referring them to a headache specialist, particularly in low-income countries, where the prevalence may even be underestimated [4]. Consequently, only a portion of migraineurs are correctly treated for their condition, while the others still suffer and are exposed to the harm and side effects of improper medications or their improper use. When a mistreated migraine patient starts using more and more painkillers to bear with her recurrent pain episodes, a secondary condition may overlap, called medication overuse headache (MOH) [1]. From a clinical point of view, MOH is a different headache that shows non-specific features but worsens a pre-existing chronic (i.e., presenting at least 15 days per month) primary headache. This acute drug overuse is often linked to behavioural factors such as the anticipatory fear of the next headache attack, but it even shows some features that resemble drug addiction [5,6].

Migraine diagnosis has always constituted a challenge for the medical community since it relies entirely on the patient’s reported history in the absence of any unequivocal diagnostic biomarker to be used in everyday clinical practice. This may be one of the reasons why this widespread neurological disorder still lacks dignity in terms of healthcare costs, reimbursement, and absence from work recognition in most countries. When correctly diagnosed, migraine management relies on the use of abortive (symptomatic) drugs for acute pain control and on preventive ones to reduce headache episode frequency if necessary (i.e., when a patient complains of two or more days of debilitating headache per month) [7]. Among the acute drugs are listed generic painkillers such as paracetamol, non-steroidal anti-inflammatory drugs (NSAIDs), or specific ones such as triptans or ditans [8]. Among the preventatives, there are traditional oral drugs such as beta-blockers (e.g., atenolol, bisoprolol, metoprolol, or propranolol), antiepileptics (e.g., topiramate), angiotensin II-receptor blockers (e.g., candesartan), tricyclic antidepressants (e.g., amitriptyline), or injective ones such as onabotulinumtoxinA (BoNT-A) and the recent anti-calcitonin gene-related peptide antibodies (anti-CGRP mAbs) [7]. Unfortunately, in this broad scenario, where even non-migraine-specific drugs find their place, it becomes very challenging to find reliable elements that may predict drug efficacy a priori and may guide migraine management straightforwardly.

In recent years, the pervasive presence of artificial intelligence (AI) has become apparent, penetrating various aspects of our daily lives. Within the expansive landscape of AI, machine learning (ML) has emerged as a central and transformative subfield. Its ability to empower systems to learn through the analysis of huge amounts of data has moved it to the forefront of technological innovation. For this reason, ML systems are also called data-driven models. Moreover, within the realm of ML, deep learning stands out as a cornerstone of research and development.

Before 2006, typically, ML referred to all those non-deep learning methods. These methods, also called shallow learning methods, include algorithms that are very common nowadays, such as shallow neural networks (neural networks with only one hidden layer of nodes), random forests, support vector machines, decision trees, XGBoost, etc. [9]. However, with the introduction of depth as an architectural characteristic of models, the expansion of deep learning begins. Deep learning methods include very common architectures such as neural networks with many layers, including Convolutional Networks, Transformers, Recurrent Neural Networks, Restricted Boltzmann Machines, Deep Belief Networks, and many other architectures [10]. While deep learning has emerged as the predominant choice for addressing a myriad of tasks due to its unparalleled capacity to extract complex features from vast datasets, shallow learning approaches continue to have a certain appeal in scenarios where simplicity is paramount and data scarcity poses a constraint. The widespread adoption of these technologies, and in particular deep learning architectures, can be attributed to the substantial availability of data and extensive efforts to enhance their accessibility [11]. Consequently, AI models in medicine have demonstrated performance levels comparable to conventional diagnostic methods, indicating their potential to significantly contribute to the diagnostic process across diverse contexts.

The versatility of ML models is underscored by their ability to analyse various types of data, ranging from images and tabular data to sequences and molecular structures. For this reason, several papers have also been proposed for the study of brain diseases [12] involving the analysis of radiological images [13,14,15], histopathological images [16], spectroscopy [17], electroencephalography signal processing [18], clinical genomics [19], etc. [20]. In addition, irrespective of the nature of the data, ML excels at tackling classification tasks, such as distinguishing samples based on characteristics like malignancy, histotype, or genetics [21]. Additionally, these models prove to be valuable in detection tasks, identifying regions of interest (ROI) within images or patterns within sequences. The segmentation of data is another field where ML showcases its efficacy, along with many other contexts [15,22]. Their application also seems to have been embedded in many neurological disciplines, including epilepsy, movement disorders, neuropsychiatric diseases, etc. [23].

Despite prevailing criticisms and reservations regarding the risks associated with AI, especially in the domain of generative AI, its continued proliferation remains unabated. The accruing benefits introduced by AI systems seem to outweigh the perceived risks, contributing to the sustained growth of their adoption. Regulatory agencies have responded to this trend by acknowledging the advantages of AI and its drawbacks and initiating the formulation of pertinent regulations in this area [24,25]. Concepts like Explainable AI and Trustworthy AI have emerged as focal points in these regulatory discussions. Despite lingering concerns, the trajectory of AI adoption in medicine suggests a trend towards greater integration, supported by evolving regulations that prioritize ethical considerations and the responsible use of these advanced technologies.

In this kaleidoscopic scenario, AI could support migraine diagnosis and management in many ways. For instance, it could help non-headache specialists reach the correct diagnosis and guide them to the choice of the best treatment for the patient. The objective of this narrative review is to present the most recent evidence about the role that AI plays today in supporting the diagnosis and classification of migraine, as well as its management, including the identification of outcome measures, personalised treatments, and in therapy response prediction.

## 2. Search Methods

The review included the full original articles written in the English language of a literature search in the PubMed/Medline electronic database up to 4 December 2023. Papers presenting non-original data, such as review articles or books, have been excluded from the results but used exceptionally for introducing a new subject. The search queries used included but were not limited to “artificial intelligence migraine”, “machine learning migraine”, “AI migraine”, “artificial intelligence headache”, “machine learning headache”, and “AI headache”.

## 3. AI in Migraine Diagnosis

### 3.1. Improving Diagnosis

As already mentioned, one of the main issues associated with migraine is that it is often not recognised. Consequently, many patients spend several years not seeking medical attention and using self-medication. Moreover, general practitioners (GPs) or other non-headache specialists may come to the wrong conclusions, explaining headache with neck musculoskeletal disorders or vision defects. Therefore, patients often undergo useless additional evaluations and delay the correct diagnosis. Table 1 lists the articles that have been found that show how AI may aid clinicians in migraine diagnosis and classification. In this scenario, AI models based on questionnaires developed by headache specialists demonstrated good accuracy in recognising the correct diagnosis, especially for migraine [26]. In the second phase, such models demonstrated even the ability to increase the accuracy of the diagnoses formulated by non-headache specialists [27]. This approach could be very useful as a retest tool and could provide a better health service for patients, reducing the percentage of misdiagnoses.

Furthermore, it is possible to use hybrid intelligent approaches [28] or ML decision trees to realise systems that are able to make a reliable diagnosis of primary or secondary headache based on ICH-3 criteria [1]. The results of a cross-sectional study on 202 patients showed how a Computer-based Diagnostic Engine (CDE) proved to be a reliable tool compared to a semi-structured interview performed by a headache specialist. Particularly, CDE improved the diagnostic process, helping to rule in or out the diagnoses of migraine or probable migraine [29]. The proposed algorithm was easily implementable on any internet-connected device (e.g., computer, tablet, or mobile phone). Therefore, it is possible to think that in the future, similar tools may be used to aid GPs or non-headache specialist clinicians in reaching a correct diagnosis. Moreover, they could even be used as a pre-visit screening tool to improve and shorten the time of the patient’s visit at a headache outpatient clinic. In addition, ML models have been revealed to be even more useful for the differential diagnosis between migraine and tension-type headache (TTH), the most prevalent primary headache worldwide. In this case, it even helped to recognise the most important symptoms to distinguish the two conditions [30,31].

Migraine may be more evident among adults, but it is a very frequent disorder even in the paediatric population, with an estimated prevalence overall of around 11% [32]. The main issue for children’s diagnosis is that sometimes migraine is not so eloquent, with pain that may be shorter lasting (i.e., from 2 h). Furthermore, it may show atypical features or presentations (i.e., migraine equivalents) [33]. Here, AI may be particularly useful for the correct diagnosis, as demonstrated by a study based on headache questionnaires of children and adolescents from the ages of 6 to 17 years [34]. An AI diagnostic model based on questionnaires easily addressable to children through parents or teachers may lead to early diagnoses with less burden. Moreover, since there is a high prevalence of migraine among children, it could be easily provided to paediatricians.

A different application of AI is not related to the diagnosis of migraine itself but to the detection of its comorbidities. The relationship between migraine and a higher cardiovascular risk is long known, and it is particularly described for MwA [35]. One of the reasons may be found in the increased risk of subclinical atrial fibrillation (AF) that has been demonstrated in a large cohort of MwA patients with an AI-ECG algorithm [36]. Differently, another study tried to use ML algorithms to detect subgroups of migraine patients based on pain intensity and found even a group sharing common cervical musculoskeletal characteristics [37].

AI proved to be a useful tool even to extract real-world evidence (RWE) from electronic health records (EHRs). Traditional RWE data extraction may rely on structured information (e.g., problem lists, tick boxes, etc.), but clinicians tend to write more detailed information in free text, and the latter is harder to analyse. Here, an AI-based advanced RWE approach produced a wider number of migraine diagnoses and more accurate data [38]. This approach could be very helpful in identifying migraine patients in different contexts, both to avoid missing diagnoses and to improve data collection for research purposes.

### 3.2. Improving Classification

After a correct diagnosis, it could be useful for both clinical and research purposes to classify people affected by migraine among different subtypes (e.g., MwoA, MwA, typical aura without migraine, and familial or sporadic hemiplegic migraine). The use of artificial neural networks showed, in this case, high precision and accuracy in correctly classifying migraine patients based on their symptoms and reported characteristics [39]. In addition, resting-state EEG connectivity studied using ML methods proved to be a useful means to differentiate patients affected by MwA from MwoA [40]. Similar high-performance results in ML-based classification methods were obtained by studying resting-state magnetoencephalographic oscillatory connectivity in patients affected by CM and comparing them to healthy subjects (HS). Even the distinction with EM patients was reached with a good performance, as was the one with patients with fibromyalgia (FM) [41].

Using supervised and unsupervised ML models, it was possible to classify different types of pain in a series of migraine patients who self-reported their attacks using a mobile phone app. In the mentioned study, the authors found that the most represented pain patterns were constituted by (i) high or medium-intense, (ii) sudden, (iii) long-lasting, and (iiii) mild or low-intense pain episodes. Accordingly, they thought that the patient could even be classified into five types, one per every pain type and another one showing a mixture of pain types [42]. Such classification could improve patient management, influencing the physician’s approach based on the main pain pattern reported by the patient.

**Table 1:** Main articles using AI to improve migraine diagnosis and classification.

Reference (First Author, Year)	Type of AI	Main Results	Number of Patients/Data Analysed
Chiang, 2022 [36]	AI-ECG algorithm able to predict the risk of subclinical AF using a convolutional neural network	MwA is associated with an increased risk of subclinical AF	40,002 patients (17,840 MwA and 22,162 MwoA)
Cowan, 2022 [29]	CDE: a decision tree designed to ask questions to diagnose ICHD-3 primary headaches and several secondary ones	A positive CDE result helps rule in migraine or probable migraine diagnoses; a negative result helps rule them out	202 patients
Frid, 2019 [40]	Predictive (classification methods and attribute-selection techniques) and traditional explanatory (statistical) analyses on functional connectivity measures	Functional connectivity metrics of resting-state EEG can be considered a biomarker to differentiate MwA from MwoA; MwoA patients show higher connectivity in the theta band	52 patients (30 MwA and 22 MwoA)
Gálvez-Goicurla, 2022 [42]	Pain episodes clustered and then classified by unsupervised and supervised ML models	Migraine pain types are classified as high- or medium-intense pain episodes, sudden pain episodes, long-lasting pain episodes, and mild- or low-intense pain episodes.	344 migraine attack data from 51 patients
Hsiao, 2022 [41]	SVM algorithms to establish the classification model	Functional connectivity of resting neuromagnetic activity may identify CM; discriminative features may be found from the interactions among salience, sensorimotor, and default mode networks; the classification model exhibited excellent performance in differentiating CM from HC and high performance in distinguishing CM from EM and FM	240 subjects (70 HS, 100 CM, 35 EM, and 35 FM); data from 56 HS and 80 CM were included in the training dataset, while those of 14 HS, 20 CM, 35 EM, and 35 FM were included in the testing datasets
Katsuki, 2023 [26]	AI-based headache diagnosis model	AI model demonstrated high diagnostic performance for migraine	6058 patients (4829 with migraine, 834 with TTH, 78 with TACs, 38 with other primary headache disorders, and 279 with other headaches) (4240/6058 training and 1818/6058 test datasets)
Katsuki, 2023 [27]	AI-based headache diagnosis model	AI model improved the non-specialist diagnostic performance	4000 headache patients diagnosed by a specialist (2800 training and 1200 test datasets)
Kwon, 2020 [31]	Stacked classifier model with four layers of binary XGBoost classifiers (1. migraine vs. non-migraine; 2. TTH vs. non-TTH; 3. TAC vs. non-TAC (i.e., epicranial headaches and TCH); and 4. epicranial headaches vs. TCH)	Excellent performance of the ML approach, but good accuracy just for migraine	2162 patients who visited the headache clinic (1286 training and 876 test datasets)
Liu, 2022 [30]	Decision tree, random forest, gradient boosting algorithm, and SVM models used to build a discriminant model and a confusion matrix used to calculate the evaluation indicators of the models	Applying ML to the decision-making system for primary headaches improves diagnostic accuracy; nausea/vomiting and photophobia/phonophobia are identified as the most important factors for distinguishing migraine from TTH	173 patients (84 with migraine and 89 with TTH)
Perez-Benito, 2019 [37]	Subgrouping based on ML algorithms: nearest neighbours’ algorithm, multisource variability assessment, and random forest model	Based on pain intensity, one group of patients was younger, with lower joint positioning sense error in cervical rotation, greater cervical mobility in rotation and flexion, lower flexion-rotation test scores, positive PAIVMs reproducing migraine, normal PPTs over the tibialis anterior, a shorter migraine history, and lower cranio-vertebral angles while standing than the remaining subgroups.	67 women affected by migraine
Sanchez-Sanchez, 2021 [39]	Supervised learning technique based on an artificial neural network	Artificial neural networks can achieve high precision and accuracy in migraine classification	400 medical records of users diagnosed with pathologies associated with migraine
Sasaki, 2023 [34]	AI-based model using 17 objective items from questionnaires and predicted migraine or non-migraine diagnosis	AI model exhibited high diagnostic performance for paediatric and adolescent migraine	909 questionnaire sheets (636 training and 273 test datasets)
Simic, 2021 [28]	Various mathematical, statistical, and artificial intelligence techniques, including decision-making methodology and clustering methods	The optimal number of clusters is three, representing three classes of headaches: (i) migraine, (ii) TTH, and (iii) other primary headaches; good quality of the system	1022 subjects

Abbreviations: AI = artificial intelligence; CDE = Computer-based Diagnostic Engine; CM = chronic migraine; EM = episodic migraine; FM = fibromyalgia; HS = healthy subject; ML = machine learning; MwA = migraine with aura; MwoA = migraine without aura; PAIVMs: passive accessory intervertebral movements; PPTs: pressure pain thresholds; SVM = support vector machine; TAC = trigeminal autonomic cephalalgia; TCH = thunderclap headache; and TTH = tension-type headache.

### 3.3. Biomarker Identification

As already mentioned, one of the fields of application of AI relies on the analysis of brain imaging. Those that may look like a few magnetic resonance imaging (MRI) slices to the human eye contain plenty of information for a machine. When it comes to migraine detection, things are not easy, and a reliable biomarker has long been looked for. Table 2 shows the main papers in the literature in which AI was to try to identify a migraine biomarker. A study that used several approaches to distinguish HS from MwA patients found that the thickness of the left temporal pole, right lingual gyrus, and left pars opercularis can be considered markers for MwA. Moreover, the authors divided MwA patients between the ones with simple aura (i.e., visual—MwA-S) and the ones with complex aura (i.e., all the other aura symptoms—MwA-C). In this case, the thickness of the left pericalcarine gyrus and of the left pars opercularis represented the markers for the MwA subtype classification [43]. Other authors recognised a distinct connectome marker in MwoA patients using resting-state functional connectivity (rsFC). By using recursive feature elimination (RFE) combined with support vector machine (SVM), the authors identified a map of functional connections involving visual, default mode network, sensorimotor, and frontoparietal networks, with an accuracy ranging from 84% to 91%, helping discriminate MwoA patients from HS. Moreover, the authors identified core network alterations in migraine that were not different from those observed in chronic pain disorders such as FM and low back pain [44].

Among the biomarkers that have been proposed to recognise migraine patients, neurophysiological ones have always played a central role. It is, therefore, difficult to choose the best technique to obtain the most reliable result. A precedent study highlighted how migraine patients show an increase in the magnitude of the EEG beta band from channels T5-T3 when stimulated with flashes at 2, 4, or 6 Hz [45]. The same group took advantage of an artificial neural network model to establish which of the stimulation frequencies could be most effective in revealing the expected results [46].

A recent study attempted to distinguish CM from MOH patients or HS using an AI approach based on linear discriminant analysis and quadratic discriminant analysis. Particularly, they showed how the different groups showed different characteristics during a mental arithmetic task when comparing the features of functional near-infrared spectroscopy of the prefrontal cortex [47].

**Table 2:** Main articles using AI for migraine biomarkers identification.

Reference (First Author, Year)	Type of AI	Main Results	Number of Patients/Data Analysed
Akben, 2012 [46]	Multi-layer perceptron neural network	4 Hz of flash stimulation frequency is the most effective frequency, and an 8 s period is necessary to identify migraine at the beta band on the EEG T5-T3 channel	15 migraine patients and 15 HS
Chen, 2022 [47]	Linear discriminant analysis and quadratic discriminant analysis	The change of hemodynamic signals of HS was smaller, while there was a large difference among migraine patients	34 subjects (13 HS, 9 CM, and 12 MOH)
Mitrovic, 2023 [43]	Several models, the best being linear discriminant analysis	The thickness of the left temporal pole, right lingual gyrus, and left pars opercularis was found as markers for MwA classification; the thickness of left pericalcarine gyrus and left pars opercularis was proposed as the features for the classification between MwA-S and MwA-C	78 subjects, among which 46 MwA (22 MwA-S and 24 MwA-C) and 32 HS, with 340 different features used
Tu et al., 2020 [44]	Recursive feature elimination + SVM	Different rsFC can accurately differentiate migraine by HS. No difference in this connectome was detected between MwoA and chronic pain patients. These markers helped to predict response to acupuncture.	144 subjects, among which 70 MwoA, 46 HS, 17 CLBP, and 11 FM

Abbreviations: CLBP = chronic low back pain; CM = chronic migraine; FM = fibromyalgia; HS = healthy subject; MwA = migraine with aura; MwA-S = with simple (i.e., visual) aura; MwA-C = with complex (i.e., different or additional neurological symptoms) aura; MwoA = migraine without aura; rsFC = resting-state functional connectivity; and SVM = support vector machine.

### 3.4. Attack Prediction and Triggers

If a patient could know in advance when her migraine attack would present, she could organise her life better. Moreover, some behavioural strategies could even be effective in managing the attack and preventing it. Table 3 shows the studies that used AI to obtain models of attack forecasts. Regarding migraine attack prediction, a study demonstrated that by using data from a mobile app, it was possible to create an AI model able to predict a migraine attack the next day with good performance [48].

The question about migraine attack triggers has always been controversial and extremely personal for each patient. For some, sleep deprivation or sleep dysregulation can cause an attack, while for others, a similar causation may be found with certain foods or alcohol. Here, a Japanese group used AI to analyse big data from a large cohort of patients using a mobile phone app to note their headache attacks, together with weather changes. The population was not exclusively represented by patients affected by migraine since users were general headache sufferers, and several had no definite diagnosis. Nevertheless, the authors found a significant relationship between low barometric pressure, barometric pressure changes, high humidity, rainfall, and an increased occurrence of headache attacks [49].

## 4. AI in Migraine Management

The role of AI in migraine management has several implications, both for the prediction of MOH and the evaluation of responsiveness to acute and preventive therapies. Indeed, there is an urgent need for a personalised approach, and AI could add clinical-decision support tools for migraine. Table 4 contains the main results of the included studies.

### 4.1. The Role of AI in the Identification of Outcome Measures

The evaluation of outcomes in migraine can be challenging because of the difficulty of encapsulating subjective aspects (the so-called ‘soft’ outcomes) of the disease. For this purpose, some authors applied natural language processing (NLP) and ML algorithms, analysing 2006 encounters from 1003 patients obtained from EHRs. Eleven data elements between headache severity, severe headache descriptors, and associated symptoms (nausea, vomiting, photophobia, and phonophobia) reached an accuracy threshold >80% using the F1 score, a method used to measure the accuracy of two classifications, assuming that recall and precision are equally important. These results show that the application of AI to EHR data has high accuracy in characterising disease outcomes when compared to a score generated via manual annotation [50].

Medication overuse (MO) is one of the main contributing factors to the chronification of episodic migraine, and consequently, the prediction of MO can be useful for preventive and therapeutic purposes. Ferroni et al. (2020) applied an ML-based decision support system to a dataset of 777 consecutive migraine patients. A combined approach using SVM and Random Optimisation (RO), or RO-MO, was employed to obtain prognostic information from clinical, biochemical, drug exposure, and lifestyle data. When predicted using at least three RO-MO models, medication overuse was accurately predicted with an area under the curve (AUC) of 0.87 [51].

### 4.2. The Role of AI in Assisting the Choice of Therapy

The increasing armamentarium of antimigraine drugs has highlighted another potential field of application of AI, as recently observed in a study using big data with a two-level nested logistic regression model. In a retrospective analysis of more than 10,000,000 migraine attack records obtained using a smartphone e-diary application, the effectiveness of 25 abortive drugs for migraine attacks was tested, being significantly higher for triptans (OR, 4.8), ergots (OR, 3.02), and anti-emetics (OR, 2.67). When compared to ibuprofen, low effectiveness was observed for other medications such as acetaminophen, NSAIDs, and combination analgesics. Except for aspirin, the OR for 24 drugs achieved statistical significance, with an area under the curve (AUC) of 0.849 using the above-mentioned logistic regression model. Unfortunately, due to their relatively low use at the moment of data extraction, the effectiveness of ditans and gepants was not included in the analysis [52].

Machine prescription for CM was tested in a study including the structured clinical record of 1446 CM patients treated by 11 preventive strategies among BoNT-A, flunarizine, candesartan, serotonin noradrenaline reuptake inhibitors (SNRI), topiramate, tricyclic antidepressants (TCAs), acupuncture, valproate, beta-blockers, and serotonin agents. The authors adopted standard NLP techniques to extract information from medical records; to model individualised treatment responses, a causal multi-task Gaussian process model was implemented and validated to calculate the average treatment effects. Data obtained from individualised treatment effects show that, when compared to expert guidelines, machine prescription allowed for successful treatment in a shorter time with no significant increase in expense. Finally, logistic regression was employed to compare individualised treatment effects to the average treatment effects; higher individual response rates were observed when compared to the overall population, underlying the multifactorial pathophysiology of CM and consequent heterogeneity in responsiveness to therapies [53].

### 4.3. AI and Predicting Therapy Responses

ML approaches can also be used to predict responses to precise therapeutic strategies taken singularly. Response to anti-CGRP mAbs was evaluated in a recent multicentre study involving 712 patients (84% CM) using variables commonly recorded in real clinical practice, such as frequency of headaches, migraine days per month, and the Head Impact Test-6 (HIT-6) scale. ML-based models found an F1 score range of 0.70–0.97 with an AUC range of 0.87–0.98 at 6, 9, and 12 months after anti-CGRP therapy started, with a response rate ranging from 50% to 75%. Moreover, according to this ML model, none of the above-mentioned clinical characteristics significantly contributed to predicting response, suggesting that anti-CGRP therapies can be effective in different migraine populations [54].

The prediction of response to BoNT-A has already been evaluated in two different studies. In the first study, simulated annealing (SA) and a random tree algorithm were used to predict the response to BoNT-A using HIT-6 and several other classifiers and clusters. A total of 173 patients were included, and the efficacy of BoNT-A was tested before the first infiltration and 12–16 weeks after each infiltration. Although sampled from 18 out of 173 records, a strategy based on the HIT-6 achieved an accuracy of over 91%. Other classifiers (e.g., CM time evolution, drugs tested before BoNT-A, a first-grade family member with migraine, etc.) predicted responses with an accuracy of 85%, confirming literature data [55]. In another study, an ML method was used to predict treatment response after one single cycle and 12 weeks after the fourth BoNT-A cycle compared to baseline, according to the PREEMPT paradigm. Data from 145 patients (113 CM, 32 high-frequency EM, or HFEM) were analysed, applying several ML methods (artificial neural network, SVM, Adaptive Neuro-Fuzzy Inference System, and random forest). In the CM group, no clinical feature was able to discriminate between responders and non-responders, suggesting the increasing need for novel and multimodal biomarkers. However, in the HFEM group, four clinical features (i.e., age of migraine onset, opioid use, the anxiety subscore of the hospital anxiety and depression scale, and the Migraine Disability Assessment, or MIDAS) helped predict a good response to BoNT-A [56].

Prediction of biofeedback efficacy in migraine treatment was shown in a recent study using an artificial neural network (ANN) named ARIANNA. In this complex study, 20 women with a CM diagnosis were included before and after 12 sessions of biofeedback (3 sessions per week), and input layer parameter pre-treatment comprised age, MIDAS, superoxide dismutase (SOD), nitrite and nitrate (NOx), and peroxide levels. ARIANNA accurately predicted the post-treatment MIDAS score in 75%, being MIDAS correlated with NOx levels (R = −0.675) and partially with peroxide levels within a specific range (R = −0.675) [57].

In a trial exploring the effects of magnesium and cobalamin supplementation and high-intensity interval training (HIIT), clinical and biological data were collected in 60 migraine patients undergoing an AI analysis. Social network analysis aided in identifying target biomarkers for migraine, and therefore, after this analysis, CGRP was selected as a biomarker for the effect of cobalamin and magnesium treatment. The combined treatment of supplementation with aerobic exercise succeeded in reducing serum CGRP levels, MIDAS, frequency, intensity, and duration of migraine attacks. In this study, AI identified a pathophysiological and prognostic marker of migraine with a multistep computational molecular biological analysis [58].

Several lines of evidence indicate transcutaneous vagal nerve stimulation (tVNS) as having a class I recommendation for EM [59]. A recent study conducted in 70 patients with MwoA and 70 with HS using functional MRI (fMRI) showed that using an SVM, it is possible to accurately distinguish migraineurs from HS by 3650 discriminative features. In the same study, an ML-based approach was used to predict response to tVNS and identified 70 out of 3650 features located in the trigeminal cervical and rostral ventromedial medulla (TCC/RVM), thalamus, medial prefrontal cortex (mPFC), and temporal gyrus [60]. Using fMRI, a neural marker previously identified with ML methods could predict response to acupuncture [44].

Finally, a structured algorithmic analysis of 131 pain drawings using a random forest ML helped predict outcomes after headache surgery, and a poor surgical outcome (defined as a non-significant reduction in the Migraine Headache Index) was predicted by diffuse pain, facial pain, and pain at the vertex, extracted from patients’ sketches [61].

**Table 4:** Main articles using AI for migraine therapy choose and response prediction.

Reference (First Author, Year)	Type of AI	Main Results	Number of Patients/Data Analysed
Chartier et al., 2022 [61]	Random forest machine learning	Data extracted from patients’ drawings had high accuracy in defining poor surgical outcomes in headache patients.	131 pain drawings
Chiang et al., 2023 [52]	Two-level nested logistic regression model	Effectiveness of abortive drugs was accurately evaluated with this model for triptans, ergots, and anti-emetics.	10,842,795 migraine attack records extracted from an e-diary smartphone application
Ciancarelli et al., 2022 [57]	Artificial neural network called ARIANNA (artificial intelligent assistant for neural network analysis)	ARIANNA accurately predicted the post-treatment MIDAS score after biofeedback treatment in 75%.	20 women with CM
Ferroni et al., 2020 [51]	SVM and Random Optimisation (RO-MO), logistic regression	RO-MO can accurately predict medication overuse in migraine, taking into consideration clinical, biochemical, drug exposure, and lifestyle (four predictors). By using at least 3 RO-MO, accuracy can be higher than 0.87.	777 migraine patients
Fu et al., 2022 [59]	Leave-one-out cross-validation (LOOCV), SVM, and support vector regression (SVR)	3650 fMRI features accurately distinguished migraine from HS. 70 features accurately predicted response to transcranial vagal nerve stimulation (tVNS).	70 EM 70 HS
Gonzalez-Martinez et al., 2022 [54]	Classification algorithms (random forests and hyperparameters) and optimization metric (F1 score)	Independently from clinical and demographical features, AI can accurately predict responses to anti-CGRP therapies.	712 patients with migraine receiving anti-CGRP therapies
Hindiyeh et al., 2022 [50]	NLP and ML algorithms (F1 score)	Data extracted from EHR were compared to reference standards, and the average F1 score for automated extraction was 90.2% for AI for 11 features, suggesting the possibility of using AI for extracting ‘soft’ outcomes.	1003 patients 2006 encounters
Martinelli et al., 2023 [56]	Random forest, SVM, artificial neural network (ANFIS and MLP), and fuzzy clustering	AI can efficiently predict responses to BoNT-A in CM and HFEM. Only in HFEM a pattern of clinical features can predict responsiveness to BoNT-A.	113 CM 32 HFEM
Matin et al., 2022 [58]	Network and in silico analysis of differential gene expression, using STRING 11.0 database	Aerobic exercise combined with vitamin B12 and magnesium supplementation significantly ameliorated MIDAS and headache features, paralleled by a decline in CGRP levels.	60 CM
Parrales Bravo et al., 2019 [55]	Feature subset selection (C4.5, WrapperSubsetEval, and ClassifierSubsetEval), simulated annealing method (SA), and random tree	AI can predict responsiveness to BoNT-A with an accuracy ranging from 85% (using clinical data) to 91% (HIT-6).	173 CM
Stubberud et al., 2022 [53]	NLP, causal multi-task Gaussian process model, and logistic regression model	AI can help choose the right individual preventive therapy quicker.	1446 CM

Abbreviations: AI = artificial intelligence; CGRP = calcitonin gene-related peptide; CM = chronic migraine; EHR = electronic health record; EM = episodic migraine; HFEM = high-frequency episodic migraine; HS = healthy subject; ML = machine learning; NLP = natural language processing; and SVM = support vector machine.

### 4.4. Miscellaneous

The recent launch of the popular Chat Generative Pre-training Transformer (ChatGPT—i.e., an AI language model able to give answers to specific queries using reinforcement learning from human feedback) raised concern about any useful application for clinicians. A recent study tried to assess the quality of the ChatGPT replies, asking for literature papers supporting different migraine preventatives. Despite some of the answers that could have been useful, the authors reported that 66% of the provided references were fake, resulting from the so-called “hallucinations”, i.e., an inaccurate response not justified by the AI training data. So, the authors concluded that, despite the potentiality, the results of that version of ChatGPT were still unreliable for medical purposes [62].

## 5. Future Directions

The arrival of AI in the scientific world entailed nearly infinite clinical applications, both for patients and clinicians. In the first place, it would be possible to establish a correct diagnosis of migraine and to exclude, with a certain degree of certainty, other causes of headaches through an AI-based interactive questionnaire. In such a way, GPs or non-headache specialists would be supported in their work, while the patients would receive an early diagnosis and treatment, avoiding pain and exposure to unnecessary medications. The prevalence is so high, and the resources are often so few that a patient could sometimes wait even more than one year before being referred to a neurologist for suspicion of migraine. So, an AI-based provisional diagnosis could buy some precious time to correctly address the patient and the GP to the correct diagnosis and management waiting for a specialist consultation. It is worth underscoring that there will still be a role for the neurologist in the diagnostic pathway. Indeed, the patient’s examination maintains its relevance, and the exclusion of secondary headaches through the cautious identification of red flags and/or the interpretation of imaging studies is still needed [63]. Moreover, a headache expert is essential when dealing with resistant or refractory patients [64].

The scientific community has always tried to identify a reliable diagnostic biomarker of migraine, but the one to be used in everyday clinical practice is still missing. It is possible, though, that the huge amount of data that can be analysed with AI may come into use. As illustrated above, there are already protocols able to analyse imaging data from MRI and suggest migraine diagnosis or its classification. In the next few years, such techniques will become more and more accurate and available so that migraine diagnosis will not be just clinical but even supported by instrumental data.

The diffusion of wearable smart devices could be one of the main means through which AI could serve patients directly. It is indeed possible to imagine that there will be devices able to analyse internal and external factors and warn patients about an imminent migraine attack. Furthermore, if such AI-based technology could reliably detect the prodromal phase of the migraine cycle (i.e., up to 48 h before the pain starts [65]), the patient could carry out some behavioural approach (e.g., regulate sleep) to disrupt the attack. It cannot be excluded that, in the future, it would be possible to apply even pre-pain preventive pharmacological strategies.

Unfortunately, it is still not possible to know in advance which preventive treatment would be the most effective and tolerable for any migraine patient. Clinicians usually tend to choose first- or second-line preventatives based on patients’ comorbidity. It is, therefore, desirable that the AI-based analysis of clinical, neurophysiological, or imaging data could give a hint about the treatment of choice both for efficacy and tolerability. It happens that CM patients who need advanced treatments (e.g., BoNT or anti-CGRP mAbs) may wait even more than 6 months or a year in the hope of becoming responders. So, if there were algorithms able to predict the response of a treatment or the chance of conversion from non-responder to responder, clinicians’ work could be facilitated, and patients’ lives could be improved. Moreover, under the umbrella of anti-CGRP mAbs, AI may predict the best one for the patient based on clinical or paraclinical data.

## 6. Conclusions

The emerging use of AI-based approaches shows huge potential for migraine from a clinical point of view. Most applications aid clinicians in optimising the accuracy of the diagnosis using data both from electronic records and headache-designed questionnaires. Other approaches are still used nearly entirely for research purposes, such as the search for migraine biomarkers. Smartphone-based AI interfaces have shown good accuracy in migraine attack prediction, analysing both patients’ symptoms and external factors (e.g., weather). The optimisation of the choice of the preventive drug for migraine patients has always represented a challenge. Luckily, AI-based algorithms are progressively leading to advances and will soon provide tools to identify prognostic and therapeutic biomarkers, allowing more and more personalised medicine.

## Figures and Tables

**Table 3:** Main articles using AI for migraine attack forecasting.

Reference (First Author, Year)	Type of AI	Main Results	Number of Patients/Data Analysed
Katsuki, 2023 [49]	Statistical and deep learning-based methods	Low barometric pressure, barometric pressure changes, high humidity, and rainfall were associated with an increased occurrence of headache attacks	4375 filtered users with 336,951 headache events
Stubberud, 2023 [48]	Several standard ML architectures with random forest classification show the best performance	An AI migraine attack forecasting model is possible; the most predictive factors were premonitory symptoms of craving, swelling, feeling cold, the amount of sleep, and the presence and intensity of headache	18 patients who completed 388 headache diary entries

Abbreviations: AI = artificial intelligence; ML = machine learning.

## Data Availability

No new data were created or analysed in this study. Data sharing is not applicable to this article.
